# Dimethyl Fumarate Induces Metabolic Crisie to Suppress Pancreatic Carcinoma

**DOI:** 10.3389/fphar.2021.617714

**Published:** 2021-02-22

**Authors:** Kaiyuan Chen, Shanshan Wu, Sisi Ye, Huimin Huang, Yi Zhou, Hongfei Zhou, Shijia Wu, Yefan Mao, Fugen Shangguan, Linhua Lan, Bicheng Chen

**Affiliations:** ^1^ Key Laboratory of Diagnosis and Treatment of Severe Hepato-Pancreatic Diseases of Zhejiang Province, The First Affiliated Hospital of Wenzhou Medical University, Wenzhou, China; ^2^ Laboratory of Precision Medical Center, The First Affiliated Hospital of Wenzhou Medical University, Wenzhou, China

**Keywords:** dimethyl fumarate, mitochondrial dynamics, folate metabolism, covalent modification, metabolic crisie

## Abstract

Dimethyl fumarate (DMF) is an approved drug used in the treatment of multiple sclerosis (MS) and psoriasis therapy. Multiple studies have demonstrated other pharmacological activities of DMF such as an anti-cancer agent. In particular, studies have shown that DMF can modulate the NRF2/HO1/NQO1 antioxidant signal pathway and inactivate NF-κB to suppress the growth of colon and breast cancer cells, and induce cell death. In this study, we aimed to evaluate the anti-tumor activities of DMF in pancreatic cancer (PC) focusing on cell death as the predominant mechanism of response. We showed that both mitochondrial respiration and aerobic glycolysis were severely depressed following treatment with DMF and the effects could be abrogated by treatment with L-cysteine and N-acetyl-L-cysteine (NAC). Importantly, we verified that DMF induced metabolic crisis and that cell death was not related to alterations in ROS. Our data implied that MTHFD1 could be a potential downstream target of DMF identified by molecular docking analysis. Finally, we confirmed that MTHFD1 is up-regulated in PC and overexpression of MTHFD1 was negatively related to outcomes of PC patients. Our data indicate that DMF induces metabolic crisie to suppress cell growth and could be a potential novel therapy in the treatment of PC.

## Introduction

The highly inefficient drug discovery process is unable to meet the requirements for novel therapies in rapidly evolving diseases. An alternative approach is to repurpose already approved drugs with known safety profiles for the treatment of different diseases. Multiple approved drugs have been demonstrated to exhibts potential activity to treat other diseases. Pioglitazone and metformin, a drug used to treat type 2 diabetes, was recently to block the proliferation of breast cancer cells and pancreatic cancer (PC) stem cells, respectively (Sancho et al., 2015; Darash-Yahana et al., 2016). Also, the FDA-approved drug chloroquine was shown to activate normal cells expressing p53 and subsequently induce Par-4 secretion to block metastasis in tumors lacking p53 (Burikhanov et al., 2017). All of these studies highlight the importance of repurposing existing drugs in other diseases including cancer.

Dimethyl fumarate (DMF) (trade name—Tecfidera) is an analog of fumarate that has been applied in the treatment of multiple sclerosis (MS), psoriasis therapy and relapsing-remitting multiple sclerosis (RRMS) (Papadopoulou et al., 2010; Gold et al., 2012; Xu et al., 2015). DMF has also been explored in several clinical trials due to its anti-inflammatory activities. Previous studies have demonstrated that low DMF concentrations can have cytoprotective effects mediated through the NRF2/HO1/NQO1 antioxidant signaling pathway. Conversely, high concentrations of DMF have been shown to induce apoptosis in several cell types (Saidu et al., 2017).

Recently, studies have shown other pharmacological effects of DMF in multiple cancer types (Loewe et al., 2006; Yamazoe et al., 2009; Saidu et al., 2017). DMF was shown to activate the MAPK signal pathway and induce autophagy while also inactivating the NRF2/HO1/DJ-1 pathway resulting in oxidative stress and enhanced cytotoxicity in colon cancer models *in vitro* and *in vivo* (Xie et al., 2015; Saidu et al., 2017; Bennett Saidu et al., 2018). Also, in melanoma and cervical cancer, DMF induces cell cycle arrest and apoptosis (Loewe et al., 2006; Yamazoe et al., 2009; Han and Zhou, 2016).

NF-κB has been shown as an essential downstream target of DMF in breast cancer, glioblastoma and cutaneous T-cell lymphoma (CTCL) (Ghods et al., 2013; Tavallai et al., 2016; Bennett Saidu et al., 2018). More recently, DMF was demonstrated to attenuate oxaliplatin-induced peripheral neuropathy without impacting the anti-tumor activity of oxaliplatin in a rodent model (Miyagi et al., 2019). However, no systematic study has yet investigated the effects of DMF in pancreatic cancer (PC).

Previous studies showed that DMF is preferentially cytotoxic to cancer cells with KRAS mutations. In particular, KRAS*G12V overexpressing colon cancer cells are more sensitive to DMF induced cell death, ROS production, and GSH depletion (Bennett Saidu et al., 2018). The KRAS gene is a crucial oncogene that is commonly mutated in most PC subtypes and more than 80% of patients carry mutations in KRAS (Liu et al., 2016). Numerous studies have shown that the inhibition of mutant KRAS activity in PC can effectively control tumor growth resulting in cancer cell death (Ostrem et al., 2013; Buscail et al., 2020; Falcomata et al., 2020; Hong et al., 2020). It has also been demonstrated that KRAS mutations are key driver events in the metabolic reprogramming of cancer cells (Son et al., 2013; Patra et al., 2018; Dey et al., 2020). Furthermore, modulating metabolism in KRAS mutant cancers may be a potential opportunity for the development of novel detection and treatment strategies in PC (Kerr et al., 2016; Halbrook and Lyssiotis, 2017).

Recently, DMF and its metabolite monomethyl fumarate (MMF) were found to modify the active site of GAPDH and inhibit its enzyme activity to subsequently repress aerobic glycolysis and modulate cellular immune processes (Kornberg et al., 2018). These studies emphasize the importance of targeting tumor metabolism as a novel therapeutic strategy in cancer. Also, considering the essential activity of DMF in regulating immune cell metabolism, we hypothesis that DMF may also inhibit PC by modulating cellular metabolism. In the current study, we aimed to evaluate the crucial roles of DMF in regulating cellular bioenergetics and to determine the underlying mechanistic basis of these effects.

## Experimental Procedures

### Cell Lines and Cell Culture

PC cell lines PANC-1, Miapaca-2, CFPAC-1 and Patu-8988 were purchased from the Cell Bank of the Chinese Academy of Sciences (Shanghai, China). PANC-1 and Miapaca-2 cells were cultured in DMEM basal medium and CFPAC-1 and Patu-8988 were cultured in RPMI-1640 basal medium. All media were supplemented with 10% Fetal Bovine Serum (FBS, S711–001S, Uruguay, U.S.) and antibiotics (100 U/ml penicillin and 100 μg/ml streptomycin, Beyotime, Nantong, Jiangsu) at 37°C, 5% CO_2_.

### Reagents

DMF (Dimethyl fumarate, CAS No. 624-49-7) was purchased from Selleck Chemicals (Houston, Texas, U.S.). A Cell Counting Kit-8 (CCK-8, C0039), mitochondrial membrane potential assay kit with JC-1 (C2006), crystal Violet Staining Solution (C0121) and ATP Assay Kit (S0026) were obtained from Beyotime (Nantong, Jiangsu, China). An annexin V-FITC/PI cell apoptosis detection kit was purchased from BD sciences (Franklin Lake, New Jersey, USA). Antibodies and other reagents are summarized in Supplementary Tables S1, S2.

### Detection of Cell Viability

To evaluate tumor-suppressive activity of DMF, PC cell lines were seeded into 96 well cell culture plates and cultured overnight. The following day, cells were treated with a vehicle and a concentration gradient of DMF (0–800 μM) and incubated for 24 h. A CCK-8 working solution was prepared by combining 100 μl of cell culture medium with 10 μL of CCK-8 solution. Cells were incubated with 110 μl of CCK-8 working solution for 1.5 h. Finally, the relative cell viability detected at OD450 was measured using a microplate reader at a wavelength of 450 nm.

To investigate the effects of metabolites and amino acids on DMF treated cells, cells were cultured with vehicle, DMF (200 μM for PANC-1, CFPAC-1 and Patu-8988, 100 μM for Miapaca-2), DMF + OAA (5 mM), DMF + α-KG (5 mM), DMF + Succinic acid (5 mM), DMF + Methionine (1 mM) DMF + SAM (1 mM), DMF + Pyruvate sodium (2 mM), DMF + L-glutamine(2.5 mM), DMF + L-serine (2 mM), DMF + NAC (10 mM), DMF + L-cysteine (1 mM) for 24 h. Under each of these conditions, the relative cell viability was determined as previously described.

### Real-Time Cellular Analysis for Cell Proliferation

To determine real-time cell proliferation, PANC-1, Miapaca-2, CFPAC-1 and Patu-8988 cells were seeded into a RTCA plate at a density of 1 × 10^4^ cells/well and incubated overnight. Cells were then treated with vehicle or DMF (50, 100, 200 μM for PANC-1, CFPAC-1 and Patu-8988; 25, 50, 100 μM for Miapaca-2 cells) and the real time cell indices recorded using the RTCA instrument.

### Colony Formation Assay

1 × 10^3^ cells were seeded into 6-well culture plates and cultured a visible cell clone was observed. Then, cells were cultured with vehicle or doses of DMF for another 2 days. Cells were washed twice with PBS and fixed with methanol solution for 15 min. The cells were then washed three times with PBS before being incubated with crystal violet staining solution for 20 min. Finally, cells were rinsed with water to remove residual crystal violet staining solution and images of the plated captured using a camera.

### Flow Cytometry Analysis of Cell Apoptosis

Vehicle and DMF treated PC cells were digested and washed twice with ice-cold PBS. Cells were then incubated with Annexin V and 7-AAD dye solution for 20 min in the dark. The prepared samples were analyzed by flow cytometry to detected cell cycle and apoptosis. Data were analyzed and plotted using Flow Jo software (Franklin Lake, New Jersey, United States).

### Determination of Mitochondrial Membrane Potential

Vehicle and DMF pretreated cells were digested and washed twice with PBS. The cell samples were then incubated with a JC-1 probe for 20 min in the dark. Finally, samples were washed twice with 1x dye solution buffer to remove residual JC-1 followed by flow cytometry analysis. Alterations in MMP are presented as the ratio of JC-1 monomers and J-aggregates.

### Measurement of Relative ATP Levels

Vehicle and DMF treated cells were collected and lyzed for 15 min. Cells were centrifuged at 12,000 g for 5 min at 4°C and the supernatants collected. Relative ATP levels were determined according to the manufacturer’s instructions. A standard curve and the ATP concentrations of the samples were measured using a luminometer.

### Detection of Total Cellular Reactive Oxygen Species

Vehicle and DMF treated cells were collected, washed twice with PBS and incubated with DCFH-DA (10 μM) dye solution for 20 min. Cell samples were washed twice with FBS-free basal medium to remove any residual probe. Finally, samples were resuspended in 300 μl of FBS-free medium and analyzed by flow cytometry.

### Protein Extraction and Western Blotting

Cell samples were collected and lyzed at 4°C for 20 min. The cell lysates were centrifuged at 12,500 rpm for 20 min at 4°C and the supernatants collected. The protein concentrations of samples were determined using a BCA protein concentration detection kit according to the manufacturer’s instructions. A total of 20 μg of protein was added per lane and the proteins separated by SDS-PAGE. Proteins were then transferred onto 0.22 μM PVDF membranes at 100 V for 80 min. Membranes were blocked in 5% non-fat milk solution at room temperature for 1.5 h and then incubated with primary antibodies overnight at 4°C. Next, the membranes were washed with TBST for 3 × 5 min and incubated with corresponding secondary antibodies at room temperature for 1 h. Membranes were washed with TBST for 3 × 10 min and the blots incubated with ECL western blotting substrate for 1 min. Finally, the blots were visualized using an ultra-sensitive multifunctional imager (Analytik Jena Chem Studioplus85).

### Cellular Bioenergetics Detection

PANC-1 (1.2 × 10^4^ cells/well), Miapaca-2 (1.5 × 1 0^4^ cells/well), CFPAC-1 (1.5 × 10^4^ cells/well) and Patu-8988 (1.5 × 10^4^ cells/well) cells were seeded into seahorse cell culture plates. The next day cells were treated for 12 h. Cellular bioenergetics of vehicle and DMF treated cells were measured using a seahorse XFe96 analyzer (Agilent Technologies). For OCR detection, cells were washed twice with OCR assay medium (0.225 g D-glucose + 1 ml pyruvate sodium +49 ml basal medium, pH7.4) and real-time OCR was measured by subsequent injection of oligomycin, FCCP and Rotenone/Antimycin A. The final concentrations of these compounds was 1 μM. For ECAR measurements, cells were washed twice with ECAR assay medium (0.0164 g L-glutamine + 50 ml basal medium, pH7.4). Overall ECAR was detected by subsequent injection of D-glucose, oligomycin and 2-DG. The final concentrations of the indicated molecules were as follows: D-glucose (10 mM), oligomycin (1 μM) and 2-DG (100 mM). Finally, overall OCR and ECAR curves were normalized to protein concentrations and plotted using Wave software (Agilent Technologies). Each sample included at least four replicates.

### Molecular Simulation Docking

The crystal structure of MTHFD1 was downloaded from the PDB database (1A4I) (Allaire et al., 1998). Auto-dock (MGLTools-1.5.6) was performed to rigid butt and docking analysis of MTHFD1, DMF and NADPH according to the manufacturer`s protocol.

### Gene Expression Profiles and Survival Analysis

The expression of folate metabolism catalytic enzymes was plotted by Gepia 2.0 (Zhang’s Lab, Peking University). Data is shown as log_2_ (TPM+1). Samples were analyzed from TCGA (tumor and matched normal samples) and the GTEx databases. Overall survival analyses of specific molecules was performed by Kaplan Meier survival evaluation. The cutoff values for each of the groups were set as the median.

### Statistical Analysis

Data in this study were statistically analyzed using SPSS 16.0 statistical software packages (SPSS Inc., Chicago, IL, United States) and presented using Graph Pad Prism 5.0 software (Graph Pad Software, Inc., La Jolla, CA, United States). The statistical significance of the groups was analyzed using a two-sided student`s *t*-test. Data are presented as the mean ± SD or the mean ± SEM. *<*p*< 0.05, **p < 0.01, ***p < 0.001.

## Results

### Dimethyl Fumarate Suppresses PC Cell Growth

**FIGURE 1:**
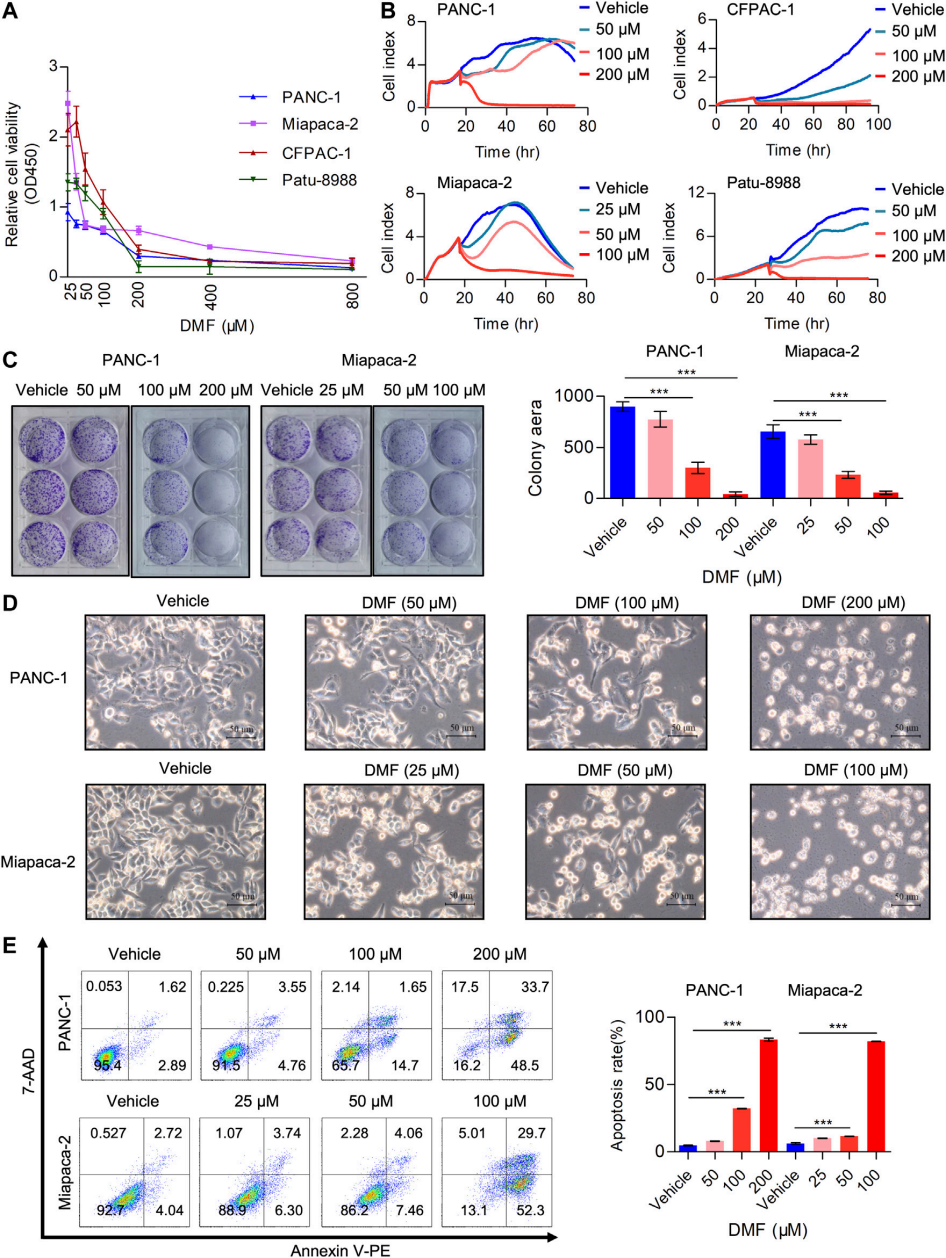
DMF inhibits pancreatic carcinoma cell proliferation and induces cell death. **(A)** Relative cell viability of PANC-1, Miapaca-2, CFPAC-1 and Patu-8988 cells treated with vehicle or gradient concentration of DMF (0, 25, 50, 100, 200, 400, 800 μM) for 24 h. **(B)** Cell index of vehicle or DMF treated cells was measured by RTCA. **(C)** Representative images of pancreatic carcinoma cell colony culture with or without DMF and quantitative analysis of colony area. **(D)** PANC-1 and Miapaca-2 cells were treated with or without DMF for 24 h, pictures were captured by Leica microscope. **(E)** Flow cytometry analysis of cell death in vehicle and DMF treated PC cells.

Multiple studies have demonstrated the anti-tumor activity of DMF in several cancer types including leukemia, colon and breast cancer (Saidu et al., 2019). However, the potential activity of DMF in PC is yet to be reported. In the current study, we aimed to evaluate the tumor-suppressive properties of DMF in PC. PC cell lines (PANC-1, Miapaca-2, CFPAC-1 and Patu-8988) were treated with a range of DMF concentrations (0–800 μM) for 24 h. We found that cell viability decreased in a DMF dose-dependent manner (Figure 1A). The inhibitory activity of DMF (50, 100, 200 μM for PANC-1, CFPAC-1 and Patu-8988; 25, 50, 100 μM for Miapaca-2 cells) on cell growth was further confirmed by RTCA and colony formation assays. Our data showed cell growth was significantly inhibited in response to DMF administration with Miapaca-2 cells being the most sensitive to DMF treatment (Figures 1B,C, Supplementary Figure S1A). Morphology changes in the vehicle and DMF treated cells were captured under a microscope showed a large number of dead cells after DMF treatment (Figure 1D, Supplementary Figure S1B). Flow cytometry data confirmed that DMF induced cell death in the four PC cell lines investigated (Figure 1E & Supplementary Figure S1C). These data further demonstrate the anti-tumor activity of DMF.

### Dimethyl Fumarate Interrupts the TCA Cycle and Depresses Mitochondrial Respiration

**FIGURE 2:**
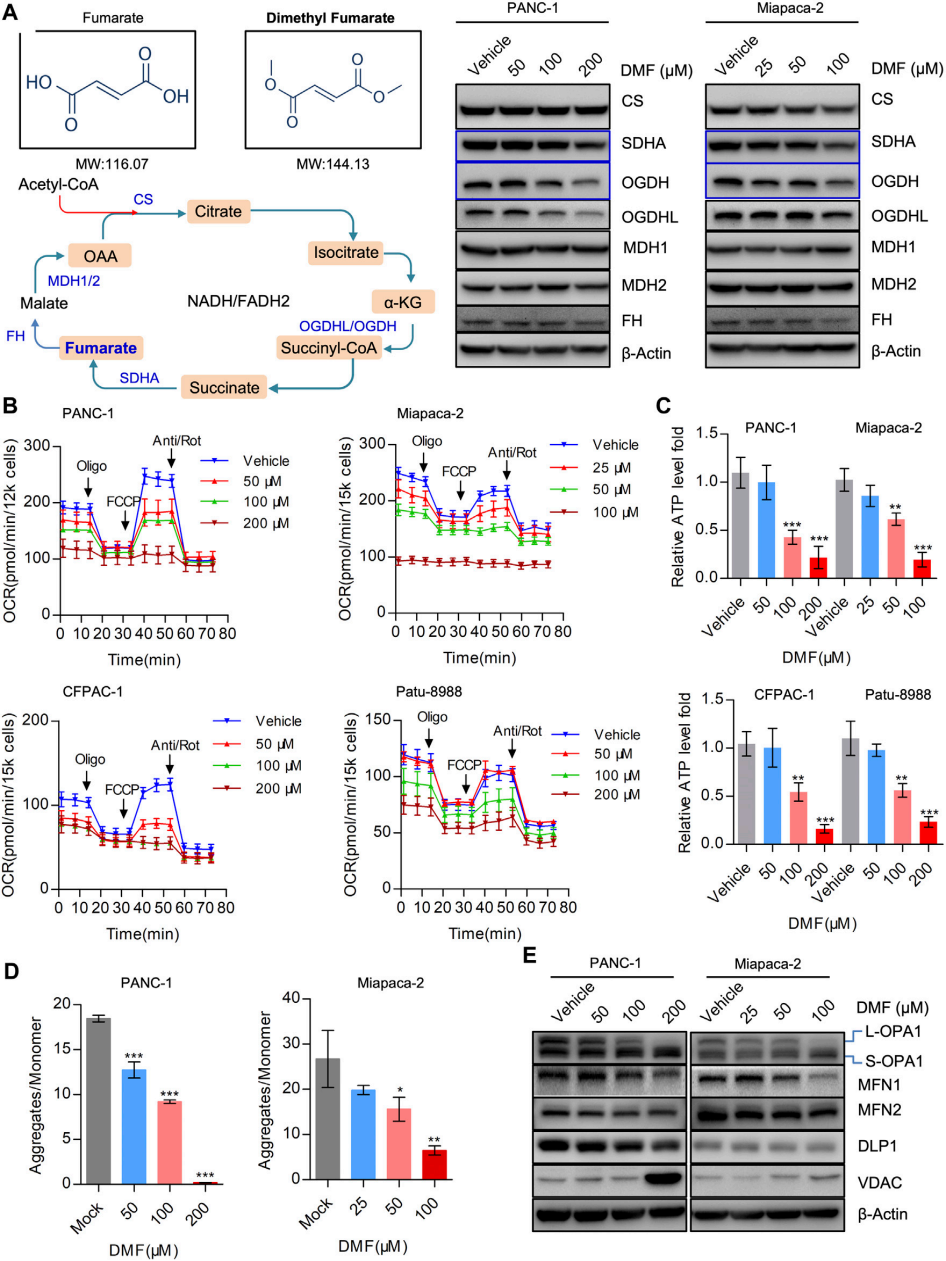
DMF severely disrupts mitochondrial respiration and imbalances mitochondrial dynamics. **(A)** Western blot analysis with antibodies against TCA cycle catalytic enzymes in vehicle and DMF treated cells. **(B)** Overall OCR alteration of vehicle and DMF (50, 100, 200 μM for PANC-1, CFPAC-1 and Patu-8988; 25, 50, 100 μM for Miapaca-2 cells) treated cells measured by seahorse XFe96 bioenergetics analyzer. **(C)** Relative ATP level of vehicle and DMF treated indicated cell lines. **(D)** MMP was represented by the ratio of JC-1 aggregates divided by monomer. **(E)** Vehicle and DMF treated PANC-1 and Miapaca-2 cells were subjected to western blot analysis with antibodies against mitochondrial dynamics associated molecules. Data are shown as Mean ± SD or Mean ± SEM. *p< 0.05, **p < 0.01, ***p < 0.001.

DMF is an immunomodulatory drug that is used to treat psoriasis and MS (Saidu et al., 2019) and can significantly suppress the proliferation of PC cells. As DMF is an analog of fumarate, we determined the levels of TCA cycle enzymes in the vehicle and DMF treated cells. We found that the levels of SDHA, OGDH and OGDHL decreased in a DMF dose-dependent manner, while no significant changes were observed in MDH1, MDH2, FH (Fumarate hydratase) and CS (Citrate synthase) (Figure 2A, Supplementary Figure S2).

It is well established that the maintenance of oxidative phosphorylation is critical for mitochondrial homeostasis. Therefore, we further measured the oxygen consumption rate (OCR) in the vehicle and DMF treated cells. Our data showed that overall OCR was significantly reduced in DMF treated cells along with basal OCR, maximal OCR and ATP production associated OCR (Figure 2B & Supplementary Figure S3). The relative ATP levels in the vehicle and DMF treated cell samples were determined and showed that ATP levels decreased with DMF treatment (Figure 2C).

We then evaluated mitochondrial membrane potential (MMP) as an index of mitochondrial homeostasis and found that DMF treatment caused a significant reduction in MMP indicating mitochondrial depolarization (Figure 2D). Also, we investigated the mitochondrial dynamics associated with molecules such as OPA1 that was cleaved from L-OPA1 to S-OPA1, while the mitochondrial fusion protein MFN1 was also shown to decrease (Figure 2E, Supplementary Figure S4). These data suggest that DMF may interrupt the TCA cycle and mitochondrial homeostasis, which subsequently induces mitochondrial depolarization and restricts mitochondrial respiration.

### Inhibition of Aerobic Glycolysis by Dimethyl Fumarate

**FIGURE 3:**
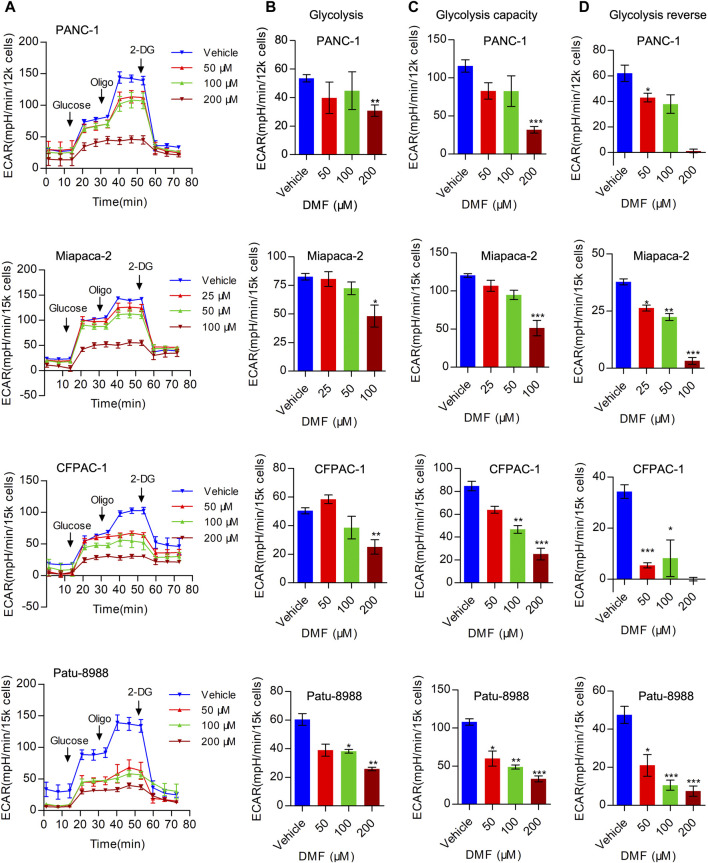
DMF represses aerobic glycolysis in pancreatic carcinoma. **(A)** PANC-1, Miapaca-2, CFPAC-1 and Patu-8988 were treated with DMF with indicated concentration for 12 h, overall ECAR was measured by seahorse XFe96 bioenergetics analyzer. **(B)** Basal glycolysis of vehicle and DMF treated cells. **(C)** Glycolysis capacity of vehicle and DMF treated cells. **(D)** Glycolysis reverse of vehicle and DMF treated cells. Data are shown as Mean ± SEM. *p< 0.05, **p < 0.01, ***p < 0.001.

In addition to mitochondrial respiration, aerobic glycolysis is considered an important process in bioenergetic metabolism that is upregulated in multiple cancer types. Next, we evaluated the overall extracellular acidification rate (ECAR) in the vehicle and DMF treated cells. We found that overall ECAR was decreased in a DMF dose-dependent manner in the four PC cell lines (Figure 3A). To further confirm the inhibitory effect of DMF on erobic glycolysis, we assessed several metabolic indices including basal glycolysis, glycolysis capacity and glycolysis reverse. Our data showed that basal glycolysis was reduced at high DMF concentrations (Figure 3B). We found that both glycolysis capacity and glycolysis reverse were decreased even with the low DMF concentration addition (Figures 3C,D). These data support the inhibitory effects of DMF on aerobic glycolysis in PC cells.

### L-cysteine and N-Acetyl-cysteine Are Efficient in Preventing Dimethyl Fumarate Induced Cell Death

**FIGURE 4:**
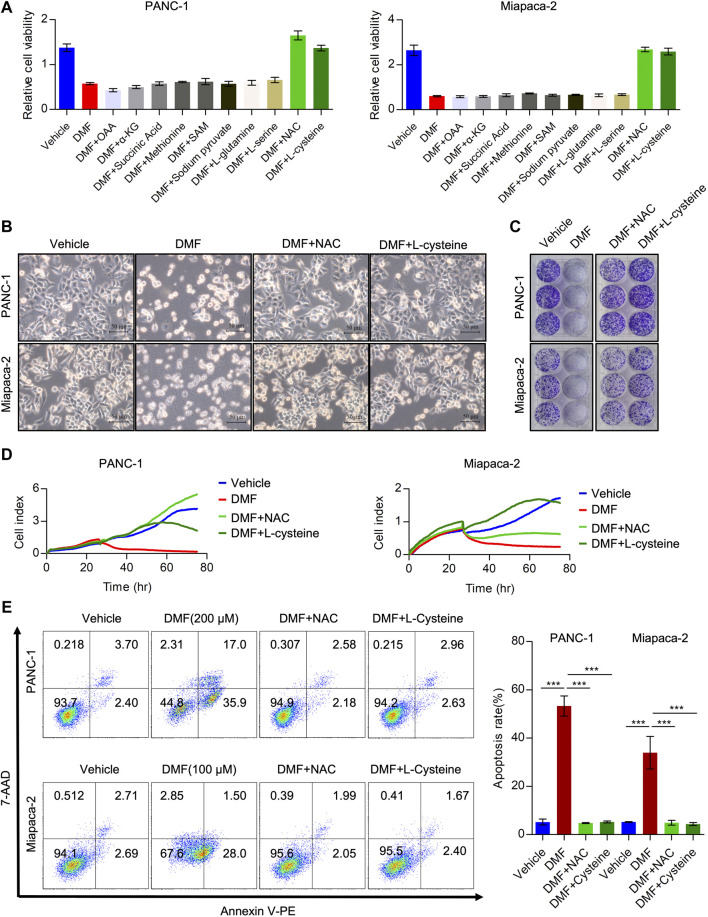
Cysteine and N-acetyl-cysteine completely abolish anti-tumor activity of DMF. **(A)** Relative cell viability of PC cells cultured with DMF or supplemented with indicated reagents. **(B)** PANC-1 andMiapaca-2cells were divided into four groups, then treated with vehicle, DMF, DMF plus NAC and DMF plus L-cysteine, respectively. Morphology changes of indicated cells were captured. (C) Representative images of vehicle, DMF, DMF plus NAC and DMF plus L-cysteine treated cell colonies. **(D)** Cell grow curve of vehicle, DMF, DMF plus NAC and DMF plus L-cysteine treated cells. **(E)** PANC-1 and Miapaca-2 cells were treated with vehicle, DMF, DMF plus NAC and DMF plus L-cysteine, respectively. 24 h later, cell apoptosis was determined by flow cytometry analysis. Data are shown as Mean ± SD.

Considering the effects of DMF on interrupting the TCA cycle and cellular bioenergetics, we hypothesized that the addition of TCA cycle metabolites could rescue these effects. To test this hypothesis, we added oxaloacetic acid (OAA), α-ketoglutaric acid (α-KG) and succinic acid to DMF treated cells and found that these metabolites could not recover the reduced cell viability induced by DMF. Moreover, we added amino acids essential for cancer cell growth and evaluated the relative cell viability to explore whether these amino acids could abolish the effects of DMF. We found that methionine, N-Acetyl-L-methionine (SAM), pyruvate sodium, L-glutamine and L-serine could not significantly rescue the reduced cell viability caused by DMF. Interestingly, we found that L-cysteine and N-Acetyl-L-cysteine (NAC) could completely restore the decreased cell viability induced by DMF (Figure 4A, Supplementary Figure S5A).

Next, we evaluated morphological changes in cells treated with vehicle, DMF, DMF + NAC and DMF + L-cysteine. We showed that supplementation with NAC and L-cysteine recovered cell death induced by DMF (Figure 4B, Supplementary Figure S5B). Furthermore, colony formation and RTCA data showed that cell growth was also rescued by NAC and L-cysteine (Figures 4C,D, Supplementary Figures S5C,D). Also, NAC and L-cysteine completely reduced DMF induced cell death (Figure 4E, Supplementary Figure S5E). These data suggest that cysteine metabolism disorders may contribute to the antitumor activity of DMF in PC.

### Cellular Bioenergetics Disorders Induced by Dimethyl Fumarate Could Be Replenished by L-cysteine and N-Acetyl-Cysteine

**FIGURE 5:**
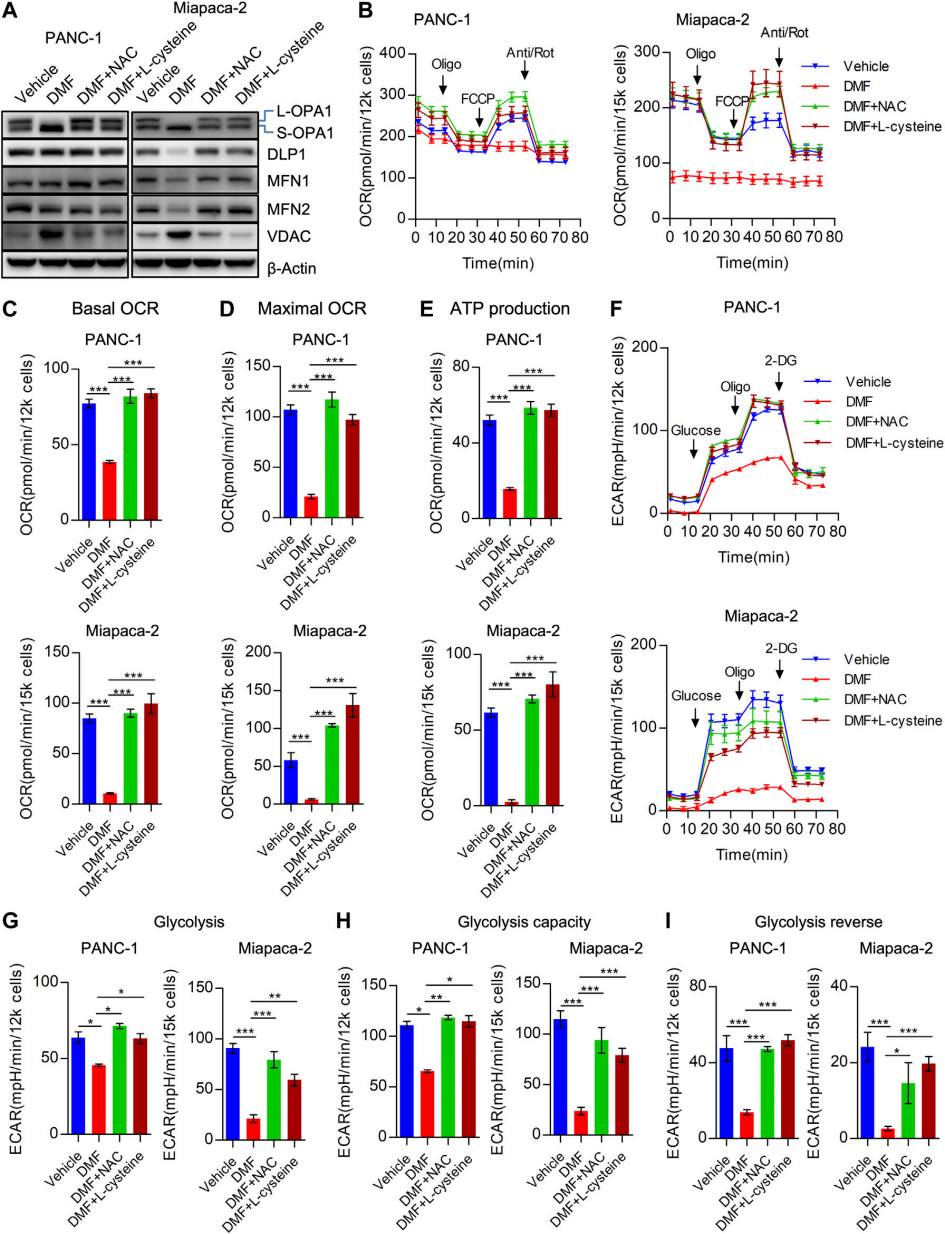
Cysteine and N-acetyl-cysteine block DMF indued cellular bioenergetics disorder. **(A)** PANC-1 and Miapaca-2 cells were treated as vehicle, DMF, DMF plus NAC and DMF plus L-cysteine, respectively. 24 h later, cell samples were collected and followed western blot analysis with indicated primary antibodies. **(B)** Overall OCR curves of vehicle, DMF, DMF plus NAC and DMF plus L-cysteine treated PANC-1 and Miapaca-2 cells. **(C)** Basal OCR to represent basal mitochondrial respiration. **(D)** Maximal OCR alteration of indicate cell groups. **(E)** ATP production associated OCR of indicate cell groups. **(F)** Overall ECAR curves of vehicle, DMF, DMF plus NAC and DMF plus L-cysteine treated PANC-1 and Miapaca-2 cells. G&H&I Basal glycolysis, glycolysis capacity and glycolysis reverse of indicate cell groups. Data are shown as Mean ± SEM. *p< 0.05, **p < 0.01, ***p < 0.001.

We determined the impact of NAC and L-cysteine on dysfunctional cellular bioenergetics induced by DMF. As predicted, we found that NAC and L-cysteine significantly abolished cleavage of OPA1 and the reduction of MFN1 indicating that deregulation of mitochondrial dynamics could be alleviated by these two molecules (Figure 5A, Supplementary Figure S6). We also found TCA cycle catalytic enzymes (SDHA, OGDH and OGDHL) were restored by NAC and L-cysteine addition to DMF treated cells (Supplementary Figure S7).

Consistently, we found that overall OCR was recovered by the addition of NAC and L-cysteine in PANC-1, Miapaca-2 and CFPAC-1 cells (Figure 5B, Supplementary Figure S8A). To further confirm the effects of NAC and L-cysteine on DMF treated cells, OCR indices were calculated. We found a reduction in basal OCR, maximal OCR and ATP associated OCR were caused by the addition of NAC and L-cysteine (Figures 5C–E, Supplementary Figures S8B–D). Similarly, we found that overall ECAR was also rescued due to the addition of NAC and L-cysteine supplement (Figure 5F, Supplementary Figure S8E). Moreover, ECAR indices including glycolysis, glycolysis capacity and glycolysis reverse were also restored in DMF + NAC and DMF + L-cysteine co-treated cells (Figures 5G–I, Supplementary Figures S8F–H).

### MTHFD1-Mediated Folate Metabolism may also be a Downstream Target of Dimethyl Fumarate

**FIGURE 6:**
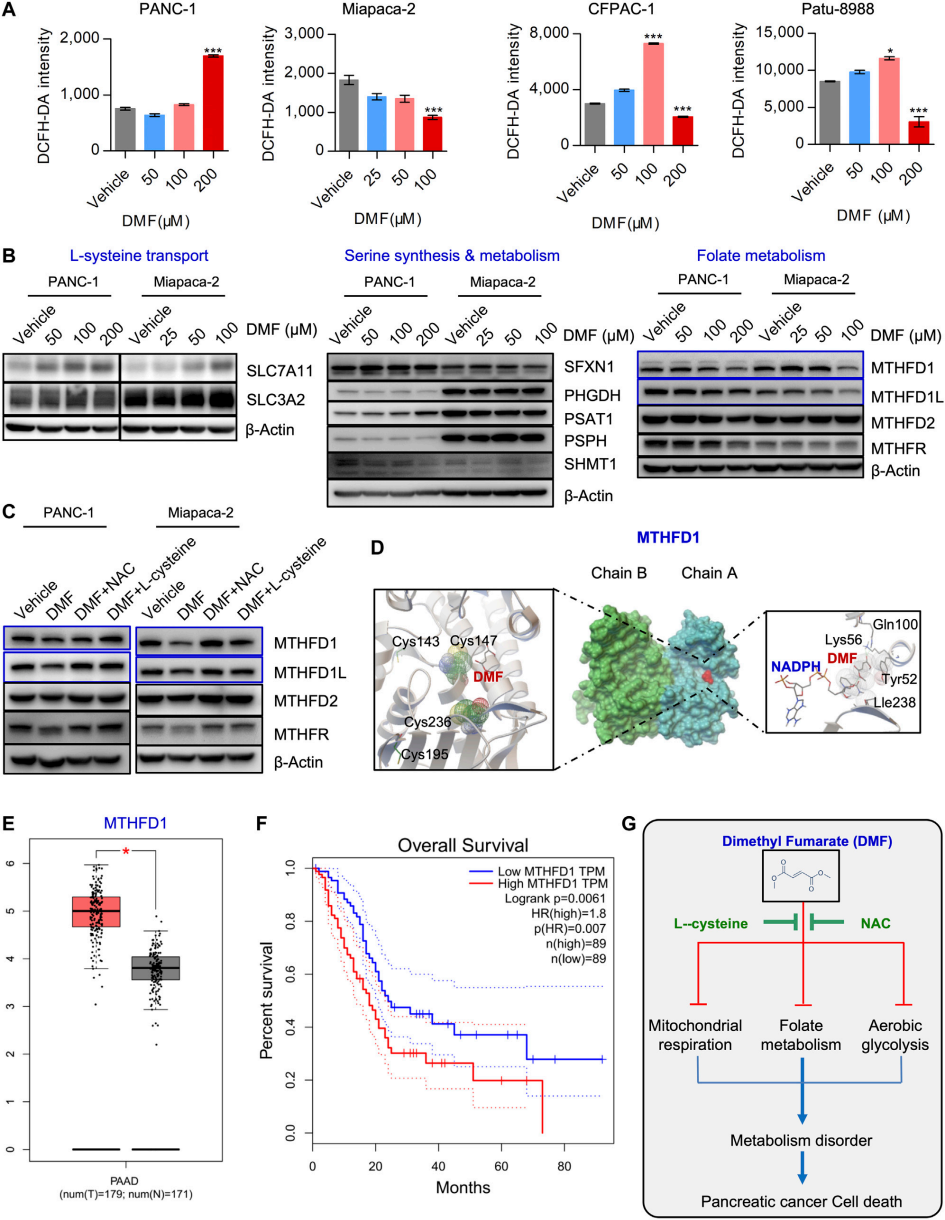
DMF also interrupts MTHFD1-MTHFD1L mediated folate metabolism. **(A)** PC cells were treated cultured with vehicle or DMF for 24 h, total cellular ROS level was measured by flow cytometry. Data are presented by fluorescence intensity of DCFH-DA. **(B)** PANC-1 and Miapaca-2 cells were treated with dosage of DMF for 24 h, followed by western blot analysis with antibodies against indicated molecules. **(C)** PANC-1 and Miapaca-2 were treated with vehicle, DMF, DMF plus NAC and DMF plus L-cysteine for 24 h, cell samples were collected and subjected to western blot analysis with primary antibodies against folate metabolism catalytic enzymes. **(D)** Pattern diagram of molecular docking about DMF and MTHFD1, NADPH. The green dotted line indicates the active hydrogen bond, mesh indicates the area of interaction, respectively. **(E)** Relative mRNA expression of MTHFD1 in non-cancerous and pancreatic carcinoma tissues. **(F)** Survival analysis of MTHFD1 expression and survival time. **(G)** Diagram of the role of DMF in inducing cell metabolism disorders and inhibiting the progression of pancreatic cancer.

Cysteine is a natural component that is used for glutathione synthesis. The derivatives of cysteine, acetylcysteine, cysteine methyl ester, cysteine acetic acid and carboxymethylcysteine, are mucolytic agents in the respiratory tract that act to promote the repair of the damaged bronchial mucosa (Bray et al., 2018; Paul et al., 2018). Our data showed that L-cysteine and N-acetyl-cysteine could perfectly rescue cell death and metabolic dysfunction induced by DMF, we hypothesized that the antitumor properties of DMF are dependent on enhanced oxidative stress. To test this hypothesis, the total cellular ROS of vehicle and DMF treated PC cells was measured. We found changes in ROS were cell-specific. ROS accumulated in a DMF dose dependent manner in PANC-1 cells, however, in the other three cell lines, low DMF concentrations induced a slight increase of ROS, while ROS rapidly decreased at high DMF concentrations (Figure 6A).

It is well known that cysteine and cysteine can be converted into each other in cells. This main source of cysteine was dependent on the transformation of intracellular serine-folate metabolism and methionine. Thus, we checked critical molecules that participate in cysteine synthesis. We found increased levels of SLC7A11, while SLC3A2 remained unchanged. Simultaneously, SFXN1, PHGDH, PSAT1, PSPH and SHMT1 were also not significantly changed. Interestingly, we found that folate metabolism enzymes such as MTHFD1 and MTHFD1L were decreased in a DMF dose-dependent manner, while no significant change was found in MTHFD2 and MTHFR (Figure 6B, Supplementary Figure S9). Also, we found that the reduction of MTHFD1 and MTHFD1L caused by DMF was restored by NAC and L-cysteine addition (Figure 6C, Supplementary Figure S10A).

Based on our molecular docking data, we found that DMF could potentially bind to the active domain of MTHFD1 and NADPH which indicated a potential regulatory property of DMF on MTHFD1 and MTHFD2.(Figure 6D, Supplementary Figure S10B). Bioinformatics analysis showed that folate metabolism catalytic enzymes such as MTHFD1 and MTHFD1L were dramatically up-regulated in PC, we also found MTHFD1 was up-regulated in PC tissues compared to pared adjacent non-cancerous tissues (Supplementary Figure S11A). However, MTHDF2, MTHFD2L and MTHFR were not significantly changed (Figure 6E, Supplementary Figure S11B). Furthermore, we found MTHFD1 expression was positively correlated with clinical stage and negatively correlated with the overall survival of PC patients (Figure 6F, Supplementary Figures S11C, S12). These data suggest that DMF inhibits PC cell growth and initiates cell death by inducing cell metabolism dysfunction (Figure 6G).

## Discussion

PC is a highly malignant disease of the digestive tract that is difficult to diagnose and treat. The 5-years survival rate is <9%, which is one of the worst prognoses of all malignant tumors (Bray et al., 2018; Siegel et al., 2019). The is a critical need for the development of novel biomarkers for the early diagnosis of PC and for novel therapeutic drugs that can improve the prognosis of patients with PC. Recently, multiple existing drugs were identified as having promising activities in diseases for which they had not been approved. This concept of drug repurposing can provide effective drugs in other diseases such as cancer. For example, pioglitazone and metformin, both of which are diabetes drugs, have shown strong antitumor activity (Sancho et al., 2015; Darash-Yahana et al., 2016). Also, the antimalarial drug hydroxychloroquine was demonstrated to overcome tamoxifen resistance in breast cancer (Lee et al., 2018). DMF, a derivative of fumaric acid, is mainly used in the treatment of relapsing multiple sclerosis (MS) and psoriasis (Hosseini et al., 2019). RecentlyDMF was shown to have potential ant-tumor properties in multiple cancer types (Saidu et al., 2019).

In the current study, we evaluated the effects of DMF in PC showing that it significantly suppresses cell growth and induces cell death. A previous study showed that DMF could induce cell apoptosis, cell cycle arrest and autophagy to suppress colorectal carcinoma cell proliferation (Kaluzki et al., 2019). DMF was also identified to induce apoptosis of hematopoietic cancer cells by inactivating NF-κB and down-regulating Bcl-XL and XIAP expression (Tsubaki et al., 2014). However, another study in colon cancer showed DMF caused GSH depletion, oxidative stress and activated MAPK signaling pathway to induce necroptosis, but not cell apoptosis (Xie et al., 2015). Interestingly, DMF was shown to induce ripoptosome-mediated death by targeting thioredoxin-1 (Schroeder et al., 2017). Therefore, the mechanism of DMF induced cell death in cancer cells remains to be fully determined. To explore this mechanism in our models, we performed a series of experiments using inhibitors of cell death pathways (caspase inhibitors Z-VAD-FMK and Z-DEVD-FMK, the RIP1 (RIPK1) inhibitor necrostatin-1, the ferroptosis inhibitors ferrostatin-1 and deferoxamine, and the ERK1/2 inhibitor SCH772984) and DMF. However, cell viability was not significantly rescued by all these inhibitors indicating that DMF-induced cell death does not occur by apoptosis, ferroptosis and necrosis (Supplementary tables S13). Further studies are needed to resolve the mechanisms of DMF induced cell death. Due to the fact that most pancreatic cancers have *KRAS* mutation, in this study, all of the four PC cell lines have *KRAS* mutation. Therefore, another important question is whether DMF could also effective to *KRAS* wild type cells or more sensitive to *KRAS* mutant PC cells, we`ll investigate this issue in our further work.

Based on the results above, we showed that DMF induced cell death and growth inhibition could be abolished by L-cysteine and NAC. Recent studies have emphasized amino acid metabolic reprogramming as a hallmark of carcinogenesis and progression (Pavlova and Thompson, 2016; Faubert et al., 2020). As a key component in the synthesis of glutathione, cysteine is crucial in maintaining redox homeostasis which contributes to cancer progression and therapeutic resistance (Bansal and simon, 2018). Recently, enhanced cysteine catabolism and serine biosynthesis have been shown to promote the production of pyruvate production during the rewiring of cellular metabolism. Furthermore, knockdown of PKM1/2 in PC cells showed that cysteine and serine metabolism is important for pancreatic carcinogenesis and progression (Yu et al., 2019).

More recently, depletion of cysteine in PC *in vitro* and *in vivo* has been shown to induce ferroptosis. This event depends on the accumulation of lipid reactive oxygen species (ROS) (Badgley et al., 2020). However, in our model, total cellular ROS levels were not significantly increased except in PANC-1 cells. Also, SLC7A11 levels increased indicating that the level of cysteine was insufficient. Combining these data with the observations that DFO and ferrostatin-1 could not effectively rescue DMF induced cell death, we suggest that DMF induced cell death is not similar to ferroptosis.

Studies have demonstrated that DMF is a well-known compound that covalently modifies cysteine residues (Blewett et al., 2016). A recent study elucidated that DMF and its metabolite monomethyl fumarate (MMF) could covalently modify the cysteine residue of GAPDH which represses erobic glycolysis and regulates immunity (Kornberg et al., 2018). Additionally, covalent modification of p65 protein caused by DMF subsequently inactivates the NF-κB signal pathway indicating a novel therapeutic strategy in advanced breast cancers (Kastrati et al., 2016). Importantly, it was shown that cell-permeable thiol N-acetyl-L-cysteine could prevent DMF causing inactivation of the NFκB pathway (Kastrati et al., 2016). These observations are similar to our work.

In our study, we found that MTHFD1 and MTHFD1L, two enzymes participating in folate metabolism, decreased upon DMF treatment which could also be recovered by NAC and L-cysteine. According to our molecular docking analysis, unexpectedly we found potential interactions between DMF and catalytic domains of MTHFD1. We also found interactions between DMF and NADPH, indicating that MTHFD1 may be a potential target of DMF. Notably, we found two cysteine residues, cys143 and cys236, were spatially close to the DMF docking domain. Several studies have shown that folate metabolic reprogramming is important for pancreatic carcinogenesis and progression (Larsson et al., 2006; Chittiboyina et al., 2018). We also found MTHFD1 depletion inhibited PC cell growth and bioenergetics (Data not shown). Therefore, further work will focus on identifying the mechanism through which DMF could also covalently modify the cysteine residues of MTHFD1 to disrupt folate metabolism. Moreover, we also found potential binding of DMF and MTHFD2. However, the expression of MTHFD2 was not significantly changed in response to DMF addition. Futher work needs to address whether DMF could affect the catalytic activity of MTHFD2.

In summary, we showed a novel mechanism of DMF in suppressing PC cell growth and inducing cell death. We demonstrated that DMF induced metabolic crisis can be reversed by L-cysteine and NAC. Also, we showed that MTHFD1 may be a potential downstream target of DMF. Overall, our data suggest that DMF could be a potential therapeutic drug for the treatment of PC.

## Data Availability

The original contributions presented in the study are included in the article/Supplementary Material, further inquiries can be directed to the corresponding authors.
