# Supplementary material for: Dimethyl Fumarate Induces Metabolic Crisie to Suppress Pancreatic Carcinoma
Source: Front Pharmacol. 2021 Feb 22;12:617714. doi: 10.3389/fphar.2021.617714 (PMC7937954; doi:10.3389/fphar.2021.617714)
Supplement: Supplementary file 1 [file datasheet1.pdf]

Supplementary materials

Dimethyl fumarate induces metabolic crisie to suppress pancreatic carcinoma

Kaiyuan Chen[1†], Shanshan Wu[1,2†], Sisi Ye[1,2†], Huimin Huang[1†], Yi Zhou[1], Hongfei Zhou[1], Shijia Wu[1], Yefan Mao[1], Fugen Shangguan[1*], Linhua Lan[1*], Bicheng Chen[1*]

[1]Key Laboratory of Diagnosis and Treatment of Severe Hepato-Pancreatic Diseases of Zhejiang Province, The First Affiliated Hospital of Wenzhou Medical University, Wenzhou 325000, P.R. China.

[2]Laboratory of Precision Medical Center, The First Affiliated Hospital of Wenzhou Medical University, Wenzhou 325000, P.R. China.

†These authors contributed equally to this work.

[*]**Correspondence**: *Bicheng Chen, email: bichengchen@hotmail.com. Linhua Lan, email: paullee90@wmu.edu.cn. Fugen Shangguan, email: mgsgfg2019@wmu.edu.cn*

Running title: DMF induces metabolic crisie.

Supplementary data

Supplementary figure 1

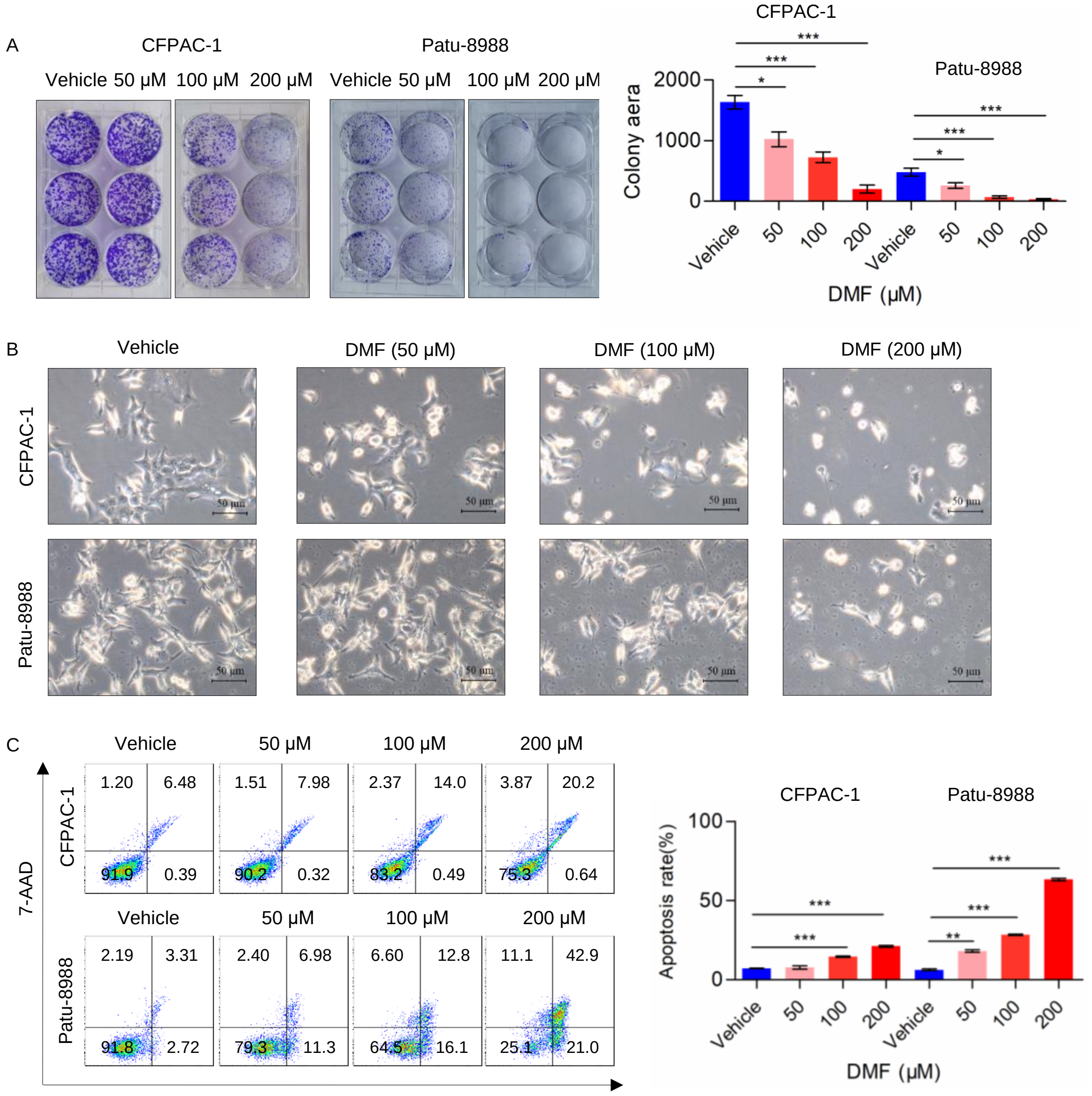

Supplementary figure 1 related to figure 1. A CFPAC-1 and Patu-8988 cells were cultured with vehicle and a gradient concentration of DMF. Cells were further stained with crystal violet, and quantitative. data of colony area was also analyzed. B CFPAC-1 and Patu-8988 cells were cultured with or without DMF for 24 hr, images were captured by Leica microscope. C Cell apoptosis analysis of vehicle and DMF treated cells. Data are shown as Mean ± SD. *<p<0.05, **p<0.01, ***p<0.001.

Supplementary figure 2

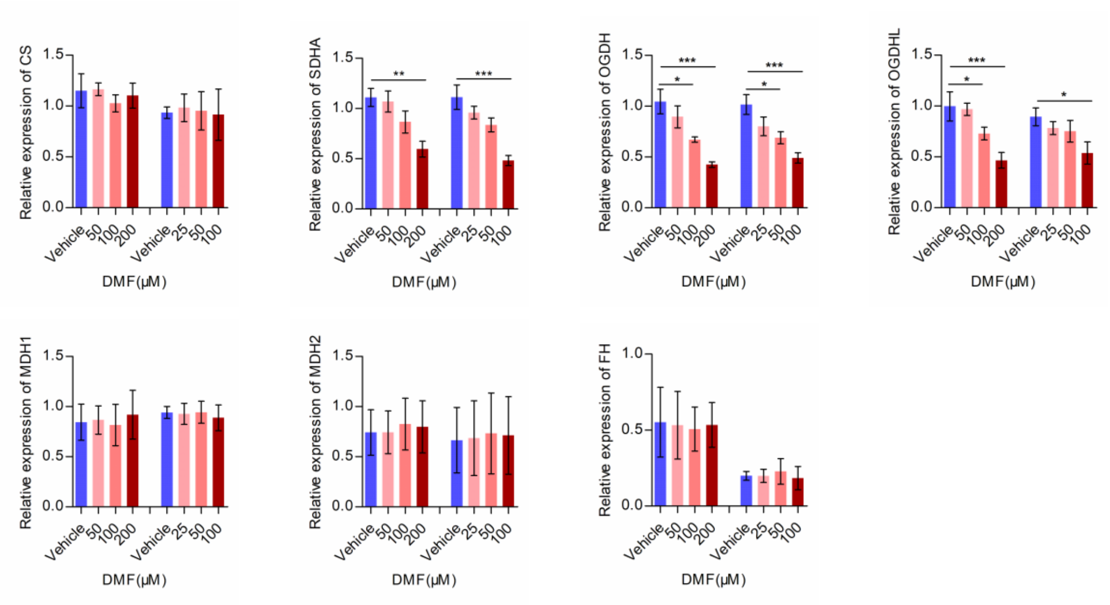

Supplementary figure 2 related to figure 2A. Quantitative analysis of protein expression in figure 2A. Data are shown as Mean ± SD. *<p<0.05, **p<0.01, ***p<0.001.

Supplementary figure 3

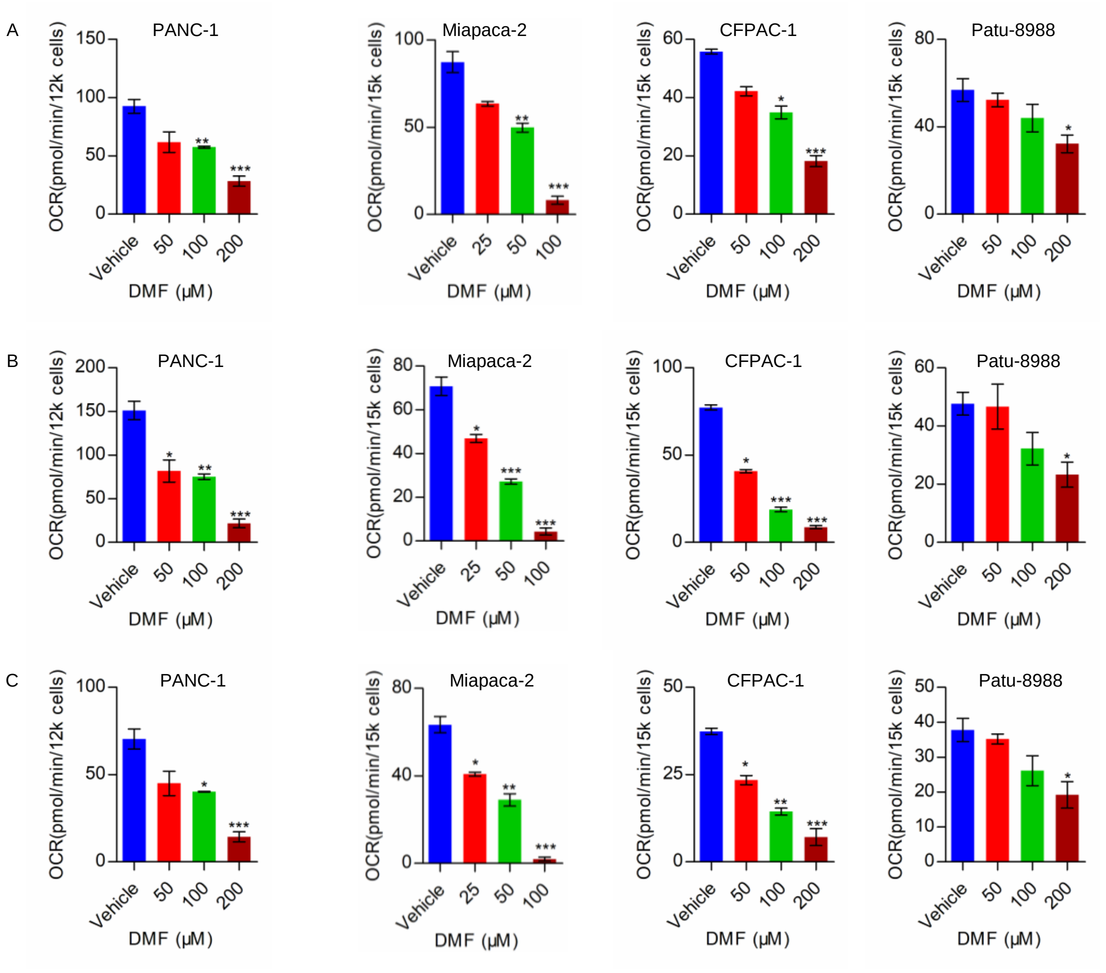

Supplementary figure 3 related to figure 2B. DMF suppresses mitochondrial respiration. A Basal OCR of vehicle and DMF treated PANC-1, Miapaca-2, CFPAC-1 and Patu-9888 cells. B Maximal OCR. C ATP production associated OCR.

Supplementary figure 4

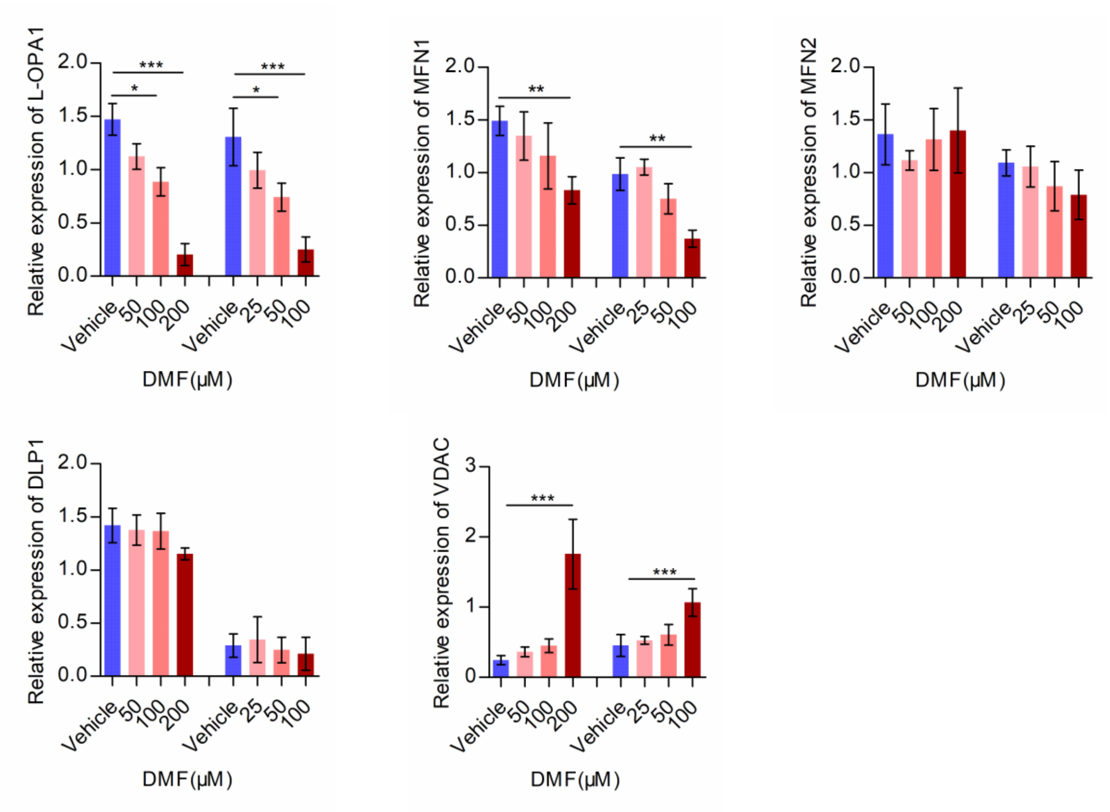

Supplementary figure 4 related to figure 2E. Quantitative analysis of protein expression in figure 2E. Data are shown as Mean ± SD. *<p<0.05, **p<0.01, ***p<0.001.

Supplementary figure 5

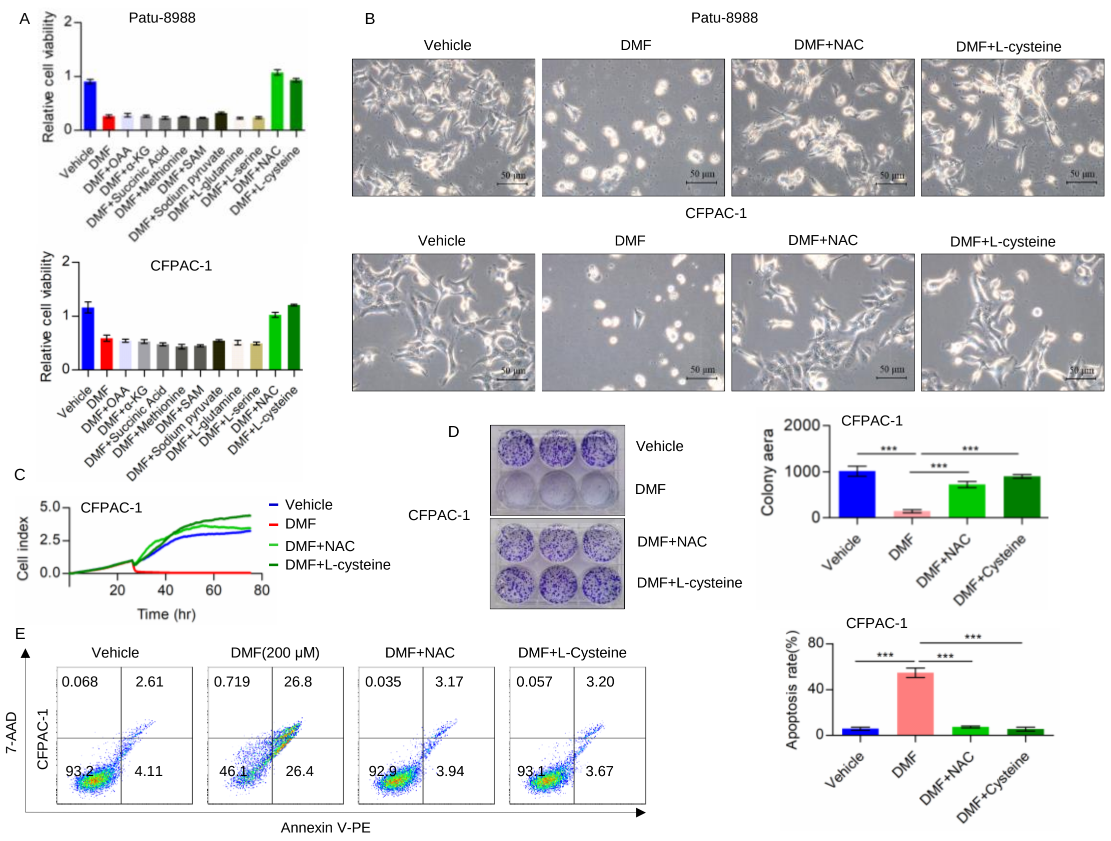

Supplementary figure 5 related to figure 4. A CFPAC-1 and Miapaca-2 cells were treated with DMF or supplemented with Oxaloacetic acid (OAA), α-Ketoglutaric acid (α-KG), Succinic acid, methionine, N-Acetyl-L-methionine (SAM), pyruvate sodium, L-glutamine, L-serine, L-cysteine and N-Acetyl-L-cysteine (NAC), relative cell viability was measured by CCK-8 kit. B Cell images of vehicle, DMF, DMF+NAC and DMF+cysteine treated cells. C&D Cell growth of CFPAC-1 treated with vehicle, DMF, DMF plus NAC and DMF plus L-cysteine were measured by RTCA and colony formation assay, respectively. E Cell apoptosis analysis of vehicle, DMF, DMF+NAC and DMF+cysteine treated cells.

Supplementary figure 6

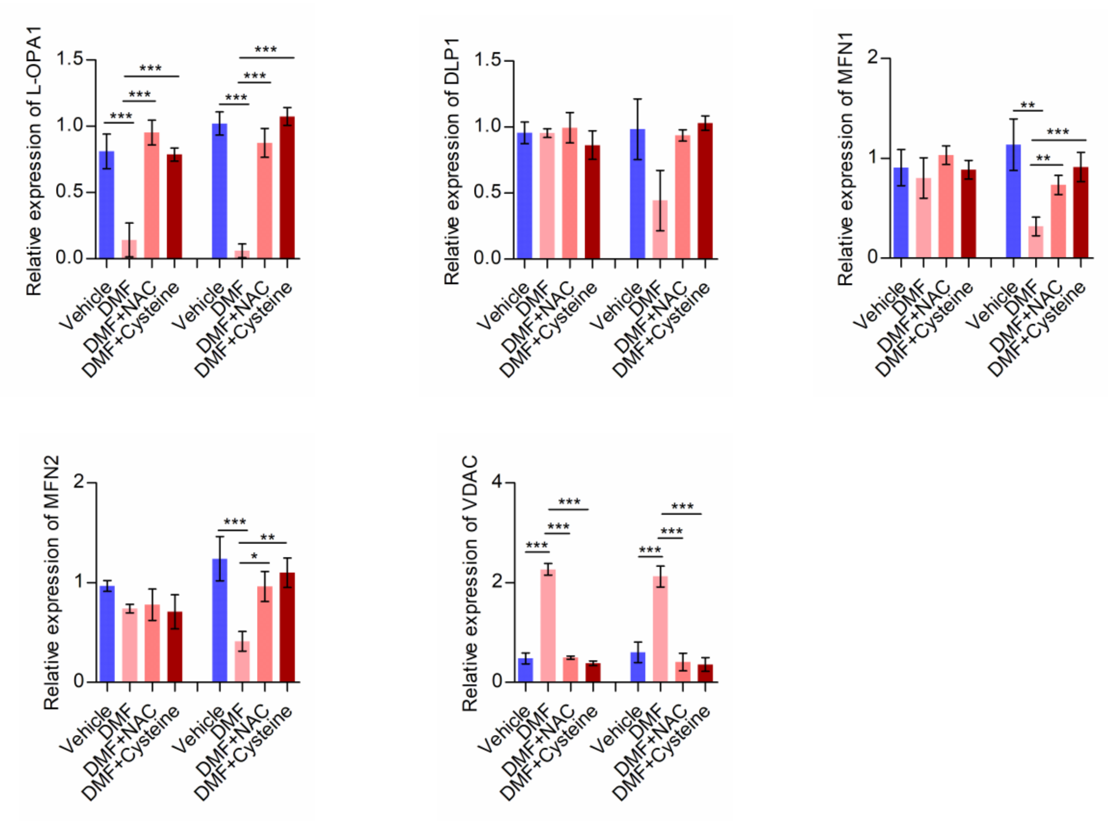

Supplementary figure 6 related to figure 5A. Quantitative analysis of protein expression in figure 5A. Data are shown as Mean ± SD. *<p<0.05, **p<0.01, ***p<0.001.

Supplementary figure 7

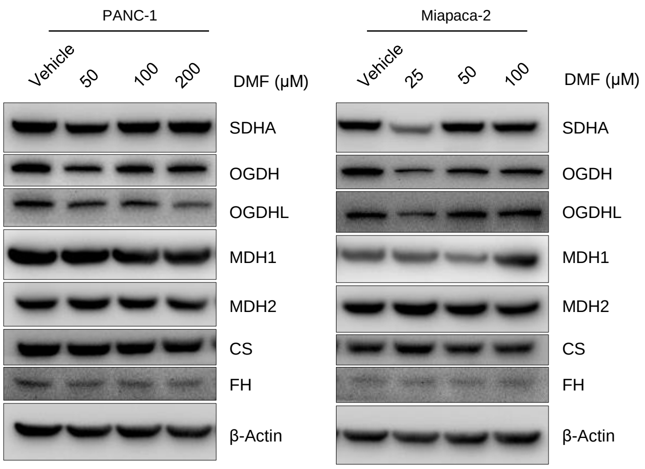

Supplementary figure 7 related to figure 5. L-cysteine and NAC supplement restore the reduction of SDHA, OGDH and OGDHL induced by DMF. PANC-1 and Miapaca-2 cells were treated with vehicle, DMF, DMF plus NAC and DMF plus L-cysteine for 24 hr. cells were collected and subjected to western blot analysis with indicated primary antibodies.

Supplementary figure 8

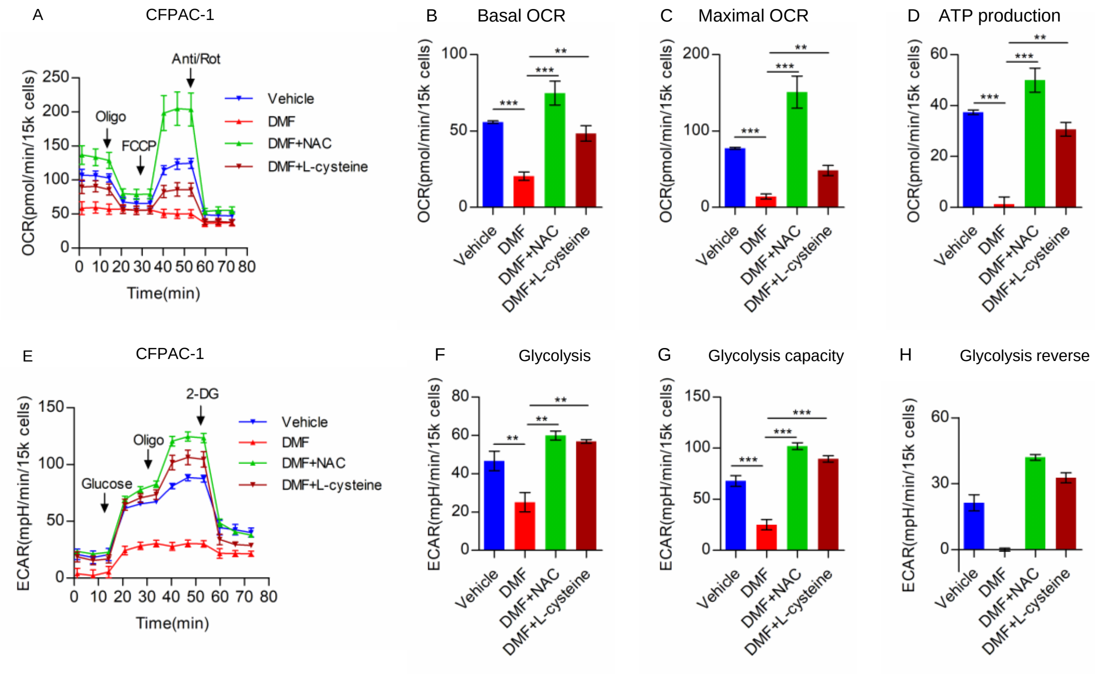

Supplementary figure 8 related to figure 5. Interruption of bioenergetics by DMF could block by L-cysteine and NAC. A Overall OCR curves of vehicle, DMF, DMF plus NAC and DMF plus L-cysteine treated CFPAC-1 cells. B Basal OCR of CFPAC-1 with indicated treatment. C Maximal OCR of CFPAC-1 cells treated with vehicle, DMF, DMF plus NAC and DMF plus L-cysteine. D ATP production associated OCR of indicate cell groups.

Supplementary figure 9

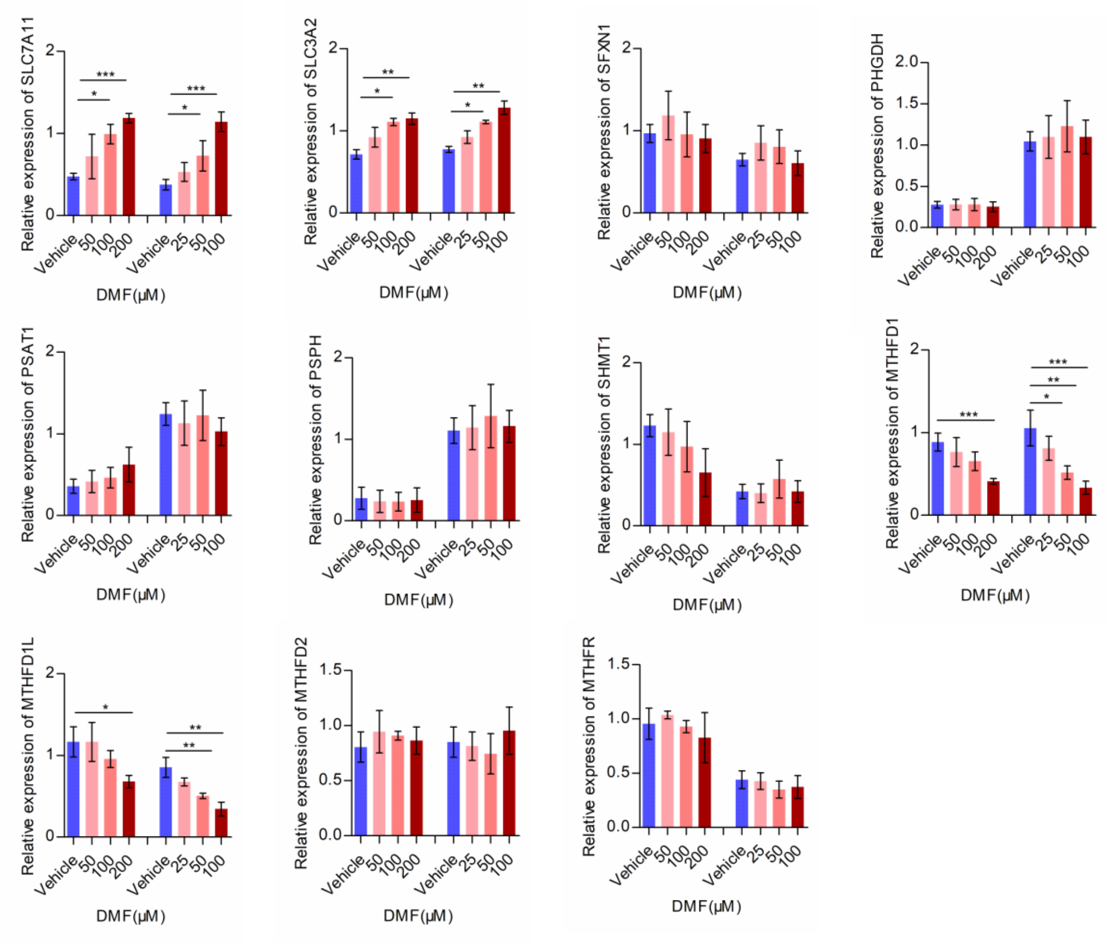

Supplementary figure 9 related to figure 6B. Quantitative analysis of protein expression in figure 6B. Data are shown as Mean ± SD. *<p<0.05, **p<0.01, ***p<0.001.

Supplementary figure 10

A

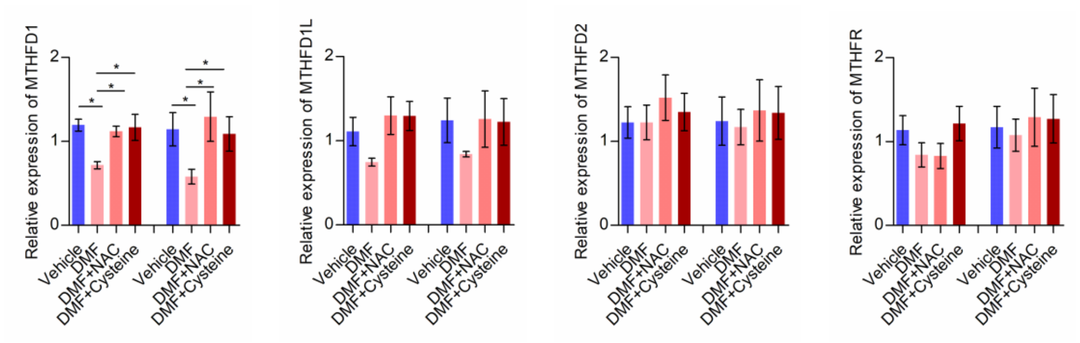

B

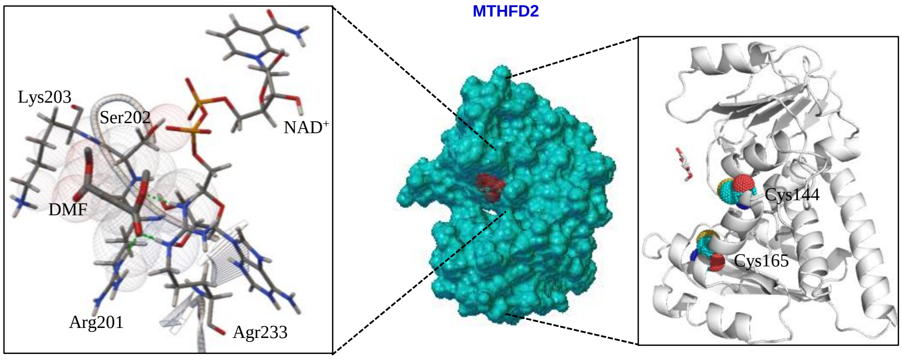

Supplementary figure 10 related to figure 6. A Quantitative analysis of protein expression in figure 6B. Data are shown as Mean ± SD. *<p<0.05. B Pattern diagram of molecular docking about DMF and MTHFD2, NADPH. The green dotted line indicates the active hydrogen bond, mesh indicates the area of interaction, respectively.

Supplementary figure 11

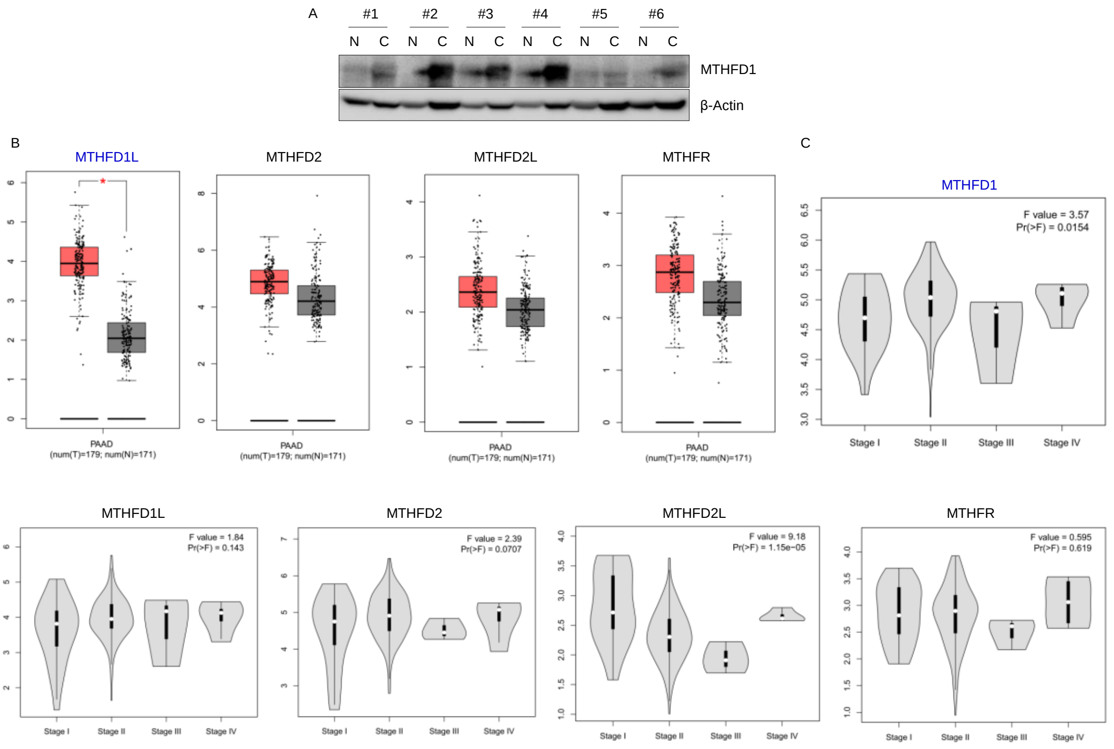

Supplementary figure 11 related to figure 6. A Protein expression of MTHFD1 in PC tissues and non-cancerous tissues. B Expression profile of indicated molecules in normal and PC tissues. C Correlation analysis of folate metabolism catalytic enzymes expression and clinical stage.

Supplementary figure 12

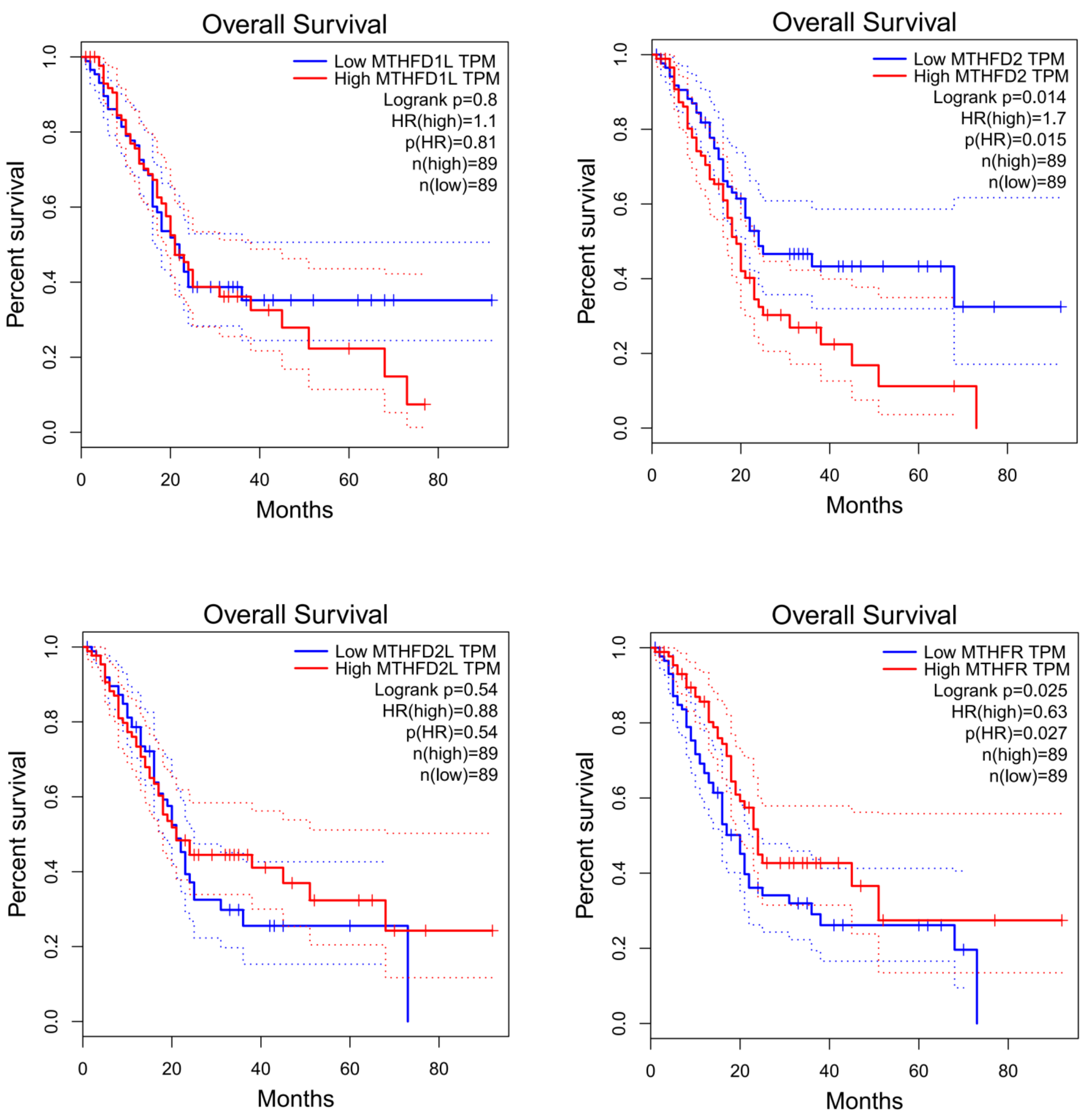

Supplementary figure 11 related to figure 6. Survival analysis of MTHFD1L, MRHFD2, MTHFD2L and MTHFR expression and outcomes of pancreatic carcinoma patients.

Supplementary figure 13

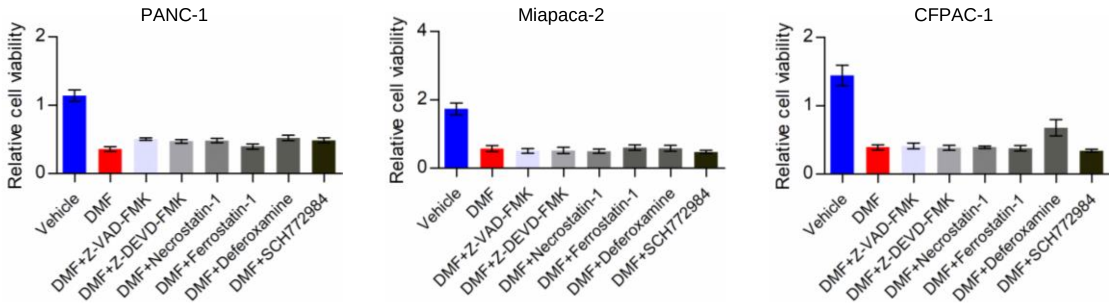

Supplementary figure 13. PANC-1, Miapaca-2 and CFPAC-1 cells were treated with DMF alone or combined with cell death inhibitors as Z-VAD-FMK (10 μM), Z-DEVD-FMK (10 μM), Necrostatin-1 (10 μM), Ferrostatin-1 (2 μM), Deferoxamine (100 μM), SCH772984 (1 μM) for 24 hr. Then, cells were incubated with CCK-8 working solution. Relative cell viability was represented by OD value at 450 nm.

Supplementary table 1

Information list of antibodies used in this study

Name	Cat No.	species	Company
SDHA	Ab14715	Mouse	Abcam
OGDH	A6391	Rabbit	ABclonal
OGDHL	A15475	Rabbit	ABclonal
MDH1	A7563	Rabbit	ABclonal
MDH2	A19906	Rabbit	ABclonal
FH (Fumarate Hydratase)	A5688	Rabbit	ABclonal
CS (Citrate Synthase)	A5713	Rabbit	ABclonal
OPA1	612607	Mouse	BD sciences
MFN1	13798-1-AP	Rabbit	Protein technology Group
MFN2	12186-1-AP	Rabbit	Protein technology Group
DLP1	611113	Mouse	BD sciences
VDAC	A11242	Rabbit	ABclonal
SLC7A11	26864-1-AP	Rabbit	Protein technology Group
SLC3A2	A5702	Rabbit	ABclonal
MTHFD1	10794-1-AP	Rabbit	Protein technology Group
MTHFD1L	16113-1-AP	Rabbit	Protein technology Group
MTHFD2	12270-1-AP	Rabbit	Protein technology Group
MTHFR	66612-1-Ig	Mouse	Protein technology Group
SFXN1	A12954	Rabbit	ABclonal
PHGDH	A10461	Rabbit	ABclonal
PSAT1	A6707	Rabbit	ABclonal
PSPH	A7924	Rabbit	ABclonal

SHMT1	A7727	Rabbit	ABclonal
β-Actin	ACO26	Rabbit	ABclonal

Supplementary table 2

Information list of reagents used in this study

Name	Cat No.	Company
Oligomycin	495455	Merck Millipore
FCCP	C2920	Sigma-Aldrich
Antimycin A	A8674	Sigma-Aldrich
Rotenone	45656	Sigma-Aldrich
2-DG	D8375	Sigma-Aldrich
D-glucose	G8270	Sigma-Aldrich
Oxaloacetic acid (OAA)	A600678	Sangon Biotech
α-Ketoglutaric acid (α-KG)	A610290	Sangon Biotech
Succinic acid	A100165	Sangon Biotech
Methionine	M2768	Sigma-Aldrich
N-Acetyl-L-methionine (SAM)	A604079	Sangon Biotech
Pyruvate sodium	P5280	Sigma-Aldrich
L-glutamine	49419	Sigma-Aldrich
L-serine	S4500	Sigma-Aldrich
N-Acetyl-L-cysteine (NAC)	S0077	Beyotime
L-cysteine	C7352	Sigma-Aldrich
Necrostatin-1	S8037	Selleck chemicals
Deferoxamine	Y0001937	Sigma-Aldrich
SCH772984	S7101	Selleck chemicals
Ferrostatin-1 (Fer-1)	S7243	Selleck chemicals
Z-VAD-FMK	HY-16658B	MedChemExpress
Z-DEVD-FMK	HY-12466	MedChemExpress
